# Supplementary material for: Resolving the full spectrum of human genome variation using Linked-Reads
Source: Genome Res. 2019 Apr;29(4):635–45. doi: 10.1101/gr.234443.118 (PMC6442396; doi:10.1101/gr.234443.118)
Supplement: Supplemental Material [file supp_gr.234443.118_Supplemental_longranger-2.2.zip › longranger-2.2.tar.bz2!/lr2.2-public/lib/stan/cmdstan/stan/doc/api/html/math_2matrix_2mdivide__right_8hpp_source.html]

Stan: src/stan/math/matrix/mdivide\_right.hpp Source File


|  |  |
| --- | --- |
|  | Stan  2.5.0  probability, sampling & optimization |


- Main Page
- Related Pages
- Namespaces
- Classes
- Files

- File List
- File Members

All Classes Namespaces Files Functions Variables Typedefs Enumerations Enumerator Friends Macros Pages

- src
- stan
- math
- matrix

mdivide\_right.hpp

Go to the documentation of this file.

1 #ifndef STAN\_\_MATH\_\_MATRIX\_\_MDIVIDE\_RIGHT\_HPP

2 #define STAN\_\_MATH\_\_MATRIX\_\_MDIVIDE\_RIGHT\_HPP

3

4 #include <boost/math/tools/promotion.hpp>

5 #include <stan/math/matrix/Eigen.hpp>

6 #include <stan/math/matrix/mdivide\_left.hpp>

7 #include <stan/math/matrix/transpose.hpp>

8 #include <stan/math/error\_handling/matrix/check\_multiplicable.hpp>

9 #include <stan/math/error\_handling/matrix/check\_square.hpp>

10

11 namespace stan {

12  namespace math {

13

22  template <typename T1, typename T2, int R1, int C1, int R2, int C2>

23  inline

24  Eigen::Matrix<typename boost::math::tools::promote\_args<T1,T2>::type,R1,C2>

25  mdivide\_right(const Eigen::Matrix<T1,R1,C1> &b,

26  const Eigen::Matrix<T2,R2,C2> &A) {

27  stan::math::check\_square("mdivide\_right(%1%)",A,"A",(double\*)0);

28  stan::math::check\_multiplicable("mdivide\_right(%1%)",b,"b",

29  A,"A",(double\*)0);

30  // FIXME: This is nice and general but likely slow.

31  return transpose(mdivide\_left(transpose(A),transpose(b)));

32 // return promote\_common<Eigen::Matrix<T1,R2,C2>,

33 // Eigen::Matrix<T2,R2,C2> >(A)

34 // .transpose()

35 // .lu()

36 // .solve(promote\_common<Eigen::Matrix<T1,R1,C1>,

37 // Eigen::Matrix<T2,R1,C1> >(b)

38 // .transpose())

39 // .transpose();

40  }

41

42  }

43 }

44 #endif

stan::math::mdivide\_right

Eigen::Matrix< typename boost::math::tools::promote\_args< T1, T2 >::type, R1, C2 > mdivide\_right(const Eigen::Matrix< T1, R1, C1 > &b, const Eigen::Matrix< T2, R2, C2 > &A)

Returns the solution of the system Ax=b.

**Definition:** mdivide\_right.hpp:25

check\_multiplicable.hpp

transpose.hpp

stan::math::check\_multiplicable

bool check\_multiplicable(const char \*function, const T1 &y1, const char \*name1, const T2 &y2, const char \*name2, T\_result \*result)

**Definition:** check\_multiplicable.hpp:15

stan::math::transpose

Eigen::Matrix< T, C, R > transpose(const Eigen::Matrix< T, R, C > &m)

**Definition:** transpose.hpp:12

Eigen.hpp

mdivide\_left.hpp

stan::math::check\_square

bool check\_square(const char \*function, const Eigen::Matrix< T\_y, Eigen::Dynamic, Eigen::Dynamic > &y, const char \*name, T\_result \*result)

Return true if the specified matrix is square.

**Definition:** check\_square.hpp:24

stan::math::mdivide\_left

Eigen::Matrix< typename boost::math::tools::promote\_args< T1, T2 >::type, R1, C2 > mdivide\_left(const Eigen::Matrix< T1, R1, C1 > &A, const Eigen::Matrix< T2, R2, C2 > &b)

Returns the solution of the system Ax=b.

**Definition:** mdivide\_left.hpp:24

check\_square.hpp


---

[ Stan Home Page ]

*© 2011–2014,
Stan Development Team.*
